# Combination of once-weekly haemodialysis with peritoneal dialysis is associated with lower mortality compared with peritoneal dialysis alone: a longitudinal study

**DOI:** 10.1093/ckj/sfaa173

**Published:** 2020-11-07

**Authors:** Miho Murashima, Takayuki Hamano, Masanori Abe, Ikuto Masakane

**Affiliations:** 1 Department of Nephrology, Graduate School of Medical Sciences, Nagoya City University, Japan; 2 Research Subcommittee of Japanese Renal Data Registry, Japanese Society for Dialysis Therapy, Japan; 3 Department of Nephrology, Osaka University Graduate School of Medicine, Japan; 4 Renal Data Registry Committee, Japanese Society for Dialysis Therapy, Japan; 5 Division of Nephrology, Hypertension and Endocrinology, Department of Internal Medicine, Nihon University School of Medicine, Japan; 6 Yabuki Hospital, Yamagata, Japan

**Keywords:** all-cause mortality, cardiovascular mortality, congestive heart failure, combination of peritoneal dialysis and haemodialysis, peritoneal dialysis

## Abstract

**Background:**

Approximately 20% of patients on peritoneal dialysis (PD) in Japan are on combination with once-weekly haemodialysis (HD). This study aimed to compare outcomes of combination therapy and PD alone.

**Methods:**

This longitudinal study on the Japanese Renal Data Registry included patients on PD from 2010 to 2014. Subjects were followed until the end of 2015. Exposure of interest was combination therapy compared with PD alone. Outcomes were complete transition to HD, all-cause mortality, cardiovascular (CV) mortality and congestive heart failure (CHF)-related mortality. Patients who initiated combination therapy were matched with those on PD alone by propensity scores. Data were analysed using Cox regression models.

**Results:**

Among the matched cohort, 608 patients were on combination therapy and 869 were on PD alone. Decline in body weight and residual renal function was more prominent in the combination therapy group. During a median follow-up of 2.5 years, 224 deaths occurred. All-cause mortality {hazard ratio (HR) [95% confidence interval (CI)] 0.56 (0.42–0.75)}, CV mortality [HR 0.48 (0.32–0.72)] and CHF-related mortality [HR 0.19 (0.07–0.55)] were significantly lower, but complete transition to HD was significantly earlier [HR 1.72 (1.45–2.03)] in the combination therapy group. Sensitivity analyses considering the effects of dialysis facilities yielded similar results. Assuming causality, numbers needed to treat to prevent one death per year were 34 patients.

**Conclusions:**

Combination therapy was associated with lower all-cause mortality, CV mortality and CHF-related mortality, but earlier transition to HD compared with PD alone, which might be due to better fluid removal by HD.

## Graphical Abstract

**Figure:**
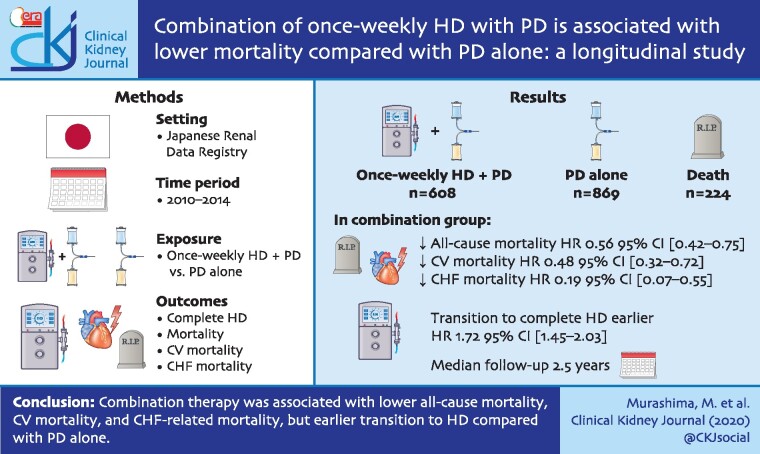

## INTRODUCTION

Combination of once-weekly haemodialysis (HD) with peritoneal dialysis (PD) is a unique type of renal replacement therapy available in Japan [1, 2]. In combination therapy (PD + HD therapy), patients usually undergo PD 5 days a week, HD once a week and no dialysis once a week, while it is a common clinical practice in Japan to perform PD 7 days a week for those on PD alone. Combination therapy intends to provide clearance and ultrafiltration which are insufficient with PD alone while maintaining a flexible lifestyle and better quality of life with PD. It was first approved by the national health insurance in 2010 in Japan, and dialysis facilities have been reimbursed for this treatment modality. The number of patients on this modality gradually increased since then [3], and by the end of 2013, 1683 patients (18.8% of PD patients) were on PD + HD therapy [4]. The guidelines published by the Japanese Society for Dialysis Therapy in 2019 stated that combination with once-weekly HD is important for those on PD with inadequate solute and fluid clearance [5].

Only a few studies have reported about outcomes of PD + HD therapy. In previous studies, after initiation of combination therapy, authors observed reduction in human atrial natriuretic peptide levels [6], improvement in blood pressure control [7], reduction in left ventricular mass index [6–8], improvement in left ventricular ejection fraction [8], reduction in hospitalization for cardiac events [8, 9] and improvement in quality of life [10, 11]. These studies suggest that PD + HD therapy may improve cardiovascular (CV) outcomes by better fluid removal by adding once-weekly HD to PD therapy. However, these studies were limited by the small number of patients enrolled and the lack of control groups. Recently, a nationwide cohort study in Japan reported that PD + HD therapy was an independent predictor of better survival [hazard ratio (HR) (95% CI): 0.60 (0.41–0.86)] [4]. However, the authors did admit that there might be an indication bias for PD + HD therapy and the duration of PD and HD among those on PD + HD was not considered in the study.

With the above background, this study aimed to compare outcomes of PD + HD therapy and PD alone using the database from the Japanese Renal Data Registry (JRDR), which is a nationwide cohort of dialysis patients in Japan. Our hypothesis was that PD + HD therapy is associated with lower all-cause mortality, CV mortality and congestive heart failure (CHF)-related mortality by better fluid removal among patients on PD + HD therapy compared with those on PD alone.

## MATERIALS AND METHODS

### Study design

This is a longitudinal study of the database from JRDR from 2010 to 2015 (SAF 2017-004). Details about JRDR were published previously [12]. In brief, it is a nationwide cohort of dialysis patients in Japan. The Japanese Society for Dialysis Therapy conducts the survey of all dialysis units in Japan at the end of every year. The response rates were 95.6%, 96.2%, 96.3%, 96.3%, 96.0% and 94.6% in 2010, 2011, 2012, 2013, 2014 and 2015, respectively. The study protocol was approved by the Medicine Ethics Committee of the Japanese Society for Dialysis Therapy, and the study was conducted in accordance with the Helsinki Declaration. The waiver of consent for JRDR was also approved by the Ethics Committee. Complete de-identification has secured the privacy of human subjects in our database, and its secondary or unofficial use (i.e. any distribution to a third party, unauthorized replication or manipulation of the database, and deviation from the proposal accepted by the Committee of Renal Data Registry) is strictly prohibited by the provision of agreements between the principal investigators and the Japanese Society for Dialysis Therapy, by which all rights regarding the database are reserved.

### Setting and participants

The inclusion criterion was undergoing PD from 2010 to 2014. The observation period terminated at the end of 2015. The exclusion criteria were age <18 years, withdrawal from dialysis or renal transplantation during the study period, unknown combination status with HD, combination of HD with peritoneal lavage only or combination of PD with HD twice or more per week. Those who transitioned from HD to PD were also excluded. Thus, included patients were those who initiated renal replacement therapy by PD, and those who switched to combination therapy or those who remained on PD alone.

### Exposure of interest and outcomes

Exposure of interest was PD + HD therapy compared with PD alone. PD + HD therapy was defined as combination of PD and once-weekly HD. Outcome variables were all-cause mortality, CV mortality and CHF-related mortality. Time to transition to HD was also compared.

### Statistical analyses

Data were shown as number (%), mean (SD), or median (interquartile range) as appropriate. Patients who initiated PD + HD therapy from 2011 to 2014 were matched with those on PD alone in the same year according to propensity scores (PSs). PS for PD + HD therapy was derived from age, sex, causes of end-stage renal disease (ESRD), PD vintage, serum blood urea nitrogen, serum creatinine, history of myocardial infarction, history of haemorrhagic stroke, history of ischaemic stroke, history of limb amputation and daily urine volume at the end of the preceding year. Patients who initiated PD + HD were matched with those on PD alone on the logit of PS (±0.25 SD). Patients who were on PD alone were matched only once (for example, those who were matched in 2011 were not matched with those who initiated PD + HD in 2012–14 even if they were on PD at the end of 2012–14). Those who initiated PD + HD therapy from 2011 to 2014 were not included in the PD alone group. All-cause mortality, CV mortality, CHF-related mortality and transition to HD were compared using Cox regression analyses. Sensitivity analyses were performed using stratified Cox regression in which data were stratified by PS-matched pairs or dialysis facilities. In addition, shared frailty model analyses were performed in which dialysis facilities were treated as a random effect. In the shared frailty models, data were adjusted for quintiles of PS. In the JRDR database, the modality of renal replacement therapy at the end of each year was available, but not the exact date when combination therapy was initiated or the exact date of transition to HD. Those on PD alone at the end of the preceding year and on PD + HD at the end of the given year were considered to initiate PD + HD therapy in the middle of the given year. Similarly, those on PD or PD + HD at the end of the preceding year and on HD at the end of the given year were considered to have transitioned to HD in the middle of the given year. Time courses of body weight and daily urine volume were compared using mixed effects models. For those on PD + HD therapy or those who transitioned to HD, post-dialysis body weight was considered. Statistical analyses were performed using Stata MP version 15.0 (Stata Corp, College Station, TX, USA).

## RESULTS

### Characteristics of patients

**FIGURE 1:**
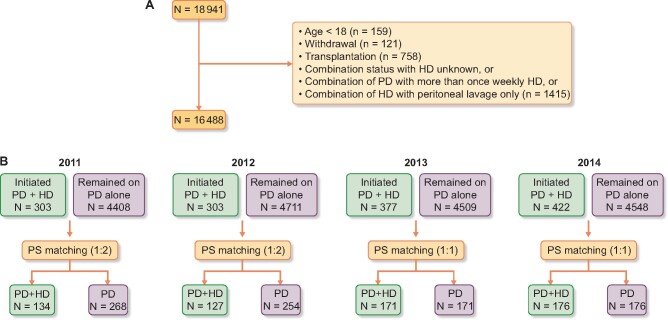
Flow diagram of patients. (**A**) Numbers of patients excluded from analyses and the reasons for exclusion are shown. Those who transited from HD to PD were excluded from the database before the standard analysis file was given to investigators. (**B**) The numbers of patients who could potentially be matched each year and the numbers of patients actually matched are shown. The same patient could be counted multiple times. For example, a patient on PD from 2011 to 2014 was included in ‘remained on PD alone’ every year. As such, the sum of PD + HD and PD patients does not match 16 488. PD + HD, combination of PD with once-weekly HD.

The flow of patients in this study is shown in Figure 1. Overall, 18 941 patients were treated with PD at some point from 2010 to 2014. After exclusion, data for 16 488 patients were available for analyses. The number of patients who initiated PD + HD therapy was 303, 303, 377 and 422 in 2011, 2012, 2013 and 2014, respectively. Patients who initiated PD + HD therapy were matched with those on PD alone by PS (1:2 in 2011 and 2012, 1:1 in 2013 and 2014). *C*-statistics for the models used to estimate PS were 0.74, 0.73, 0.76 and 0.76 in 2011, 2012, 2013 and 2014, respectively. Demographics of PS-matched cohort are shown in Table 1. The mean age was 59.3 years, 71.7% were male, ∼30% were diabetics and the median PD vintage at the end of the preceding year was 2.1 years. Demographics including blood urea nitrogen, creatinine and daily urine output were well matched. These data suggest that residual renal function was similar between the two groups.

**Table 1.** Demographics of the propensity-matched cohort

	PD + HD	PD	*d* (%)
	(*n* = 608)	(*n* = 869)	
Age	59.3 (11.6)	59.3 (13.0)	−0.1
Sex (male)	436 (71.7)	618 (71.7)	1.3
Causes of ESRD			
Glomerulonephritis	247 (40.6)	331 (38.0)	5.2
Diabetes mellitus	191 (31.4)	282 (32.5)	−2.2
Hypertension	73 (12.0)	111 (12.8)	−2.3
Others	97 (16.0)	145 (16.7)	−2.0
PD vintage (years)^a^	2.1 (1.0–4.1)	2.1 (1.0–4.0)	4.4
Blood urea nitrogen (mg/dL)	57.7 (14.4)	58.2 (15.5)	−3.6
Creatinine (mg/dL)	11.4 (3.0)	11.2 (3.1)	7.3
Urine volume (mL/day)^a^	500 (100–915)	500 (145–1000)	−8.0
History of myocardial infarction			
Yes	44 (7.2)	68 (7.8)	−2.2
No	508 (83.6)	727 (83.7)	−0.3
Unknown	56 (9.2)	74 (8.5)	2.4
History of haemorrhagic stroke			
Yes	12 (2.0)	10 (1.2)	6.6
No	542 (89.1)	785 (90.3)	−3.9
Unknown	54 (8.9)	74 (8.5)	1.3
History of ischaemic stroke			
Yes	59 (9.7)	94 (10.8)	−3.7
No	496 (81.6)	705 (81.1)	1.2
Unknown	53 (8.7)	70 (8.1)	2.4
History of limb amputation			
Yes	7 (1.2)	8 (0.9)	2.3
No	548 (90.1)	792 (91.1)	−3.5
Unknown	53 (8.7)	69 (7.9)	2.8

The data were shown in number (%), mean (SD) or median (interquartile range).

a Standardized differences were calculated after log-transformation.

PD + HD, combination of PD with once-weekly HD, *d*: standardized difference.

### Comparison of all-cause mortality, CV mortality and CHF-related mortality

**FIGURE 2:**
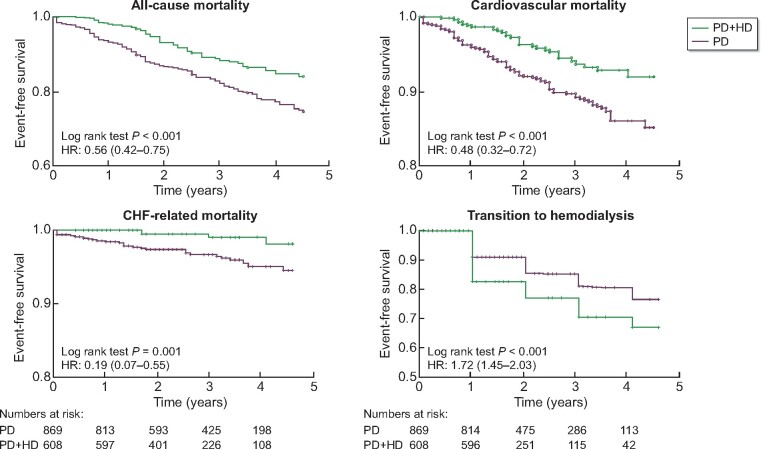
Comparison of all-cause mortality, CV mortality, CHF-related mortality and transition to HD among PS-matched cohort. Time 0 indicated the time when combination therapy was started for PD + HD patients and the time of matching for PD alone patients. The numbers at risk were shown below the graphs. The numbers at risk were the same for all-cause, CV and CHF-related mortality. PD + HD, combination of PD with once-weekly HD.

**FIGURE 3:**
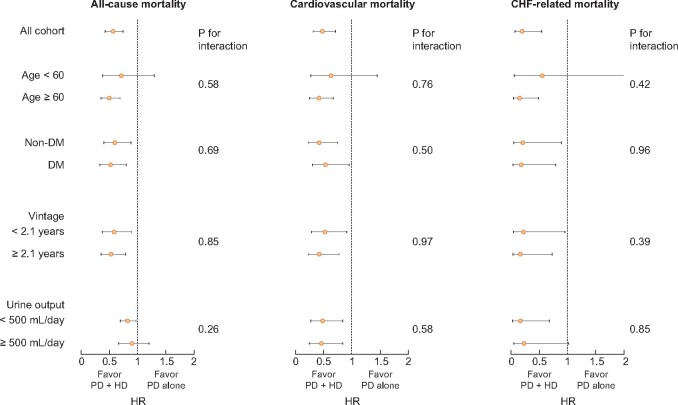
Subgroup analyses for association between PD + HD therapy with mortality. PD + HD, combination of PD with once-weekly HD; DM, diabetes mellitus.

With a median follow-up of 2.5 years, there were 61 and 163 deaths, 30 and 93 CV deaths, and 4 and 31 deaths due to CHF among those on PD + HD therapy and those on PD alone, respectively. All-cause mortality [HR 0.56 (0.42–0.75)], CV mortality [HR 0.48 (0.32–0.72)] and CHF-related mortality [HR 0.19 (0.07–0.55)] were significantly lower in the combination therapy group (Figure 2). Assuming causality, the numbers needed to treat to prevent one death, CV death and death due to CHF per year were 34, 51 and 98 patients, respectively. No effect modifications were found by age, diabetic status, dialysis vintage or daily urine volume (Figure 3).

### Comparison of the time to complete transition to HD

**FIGURE 4:**
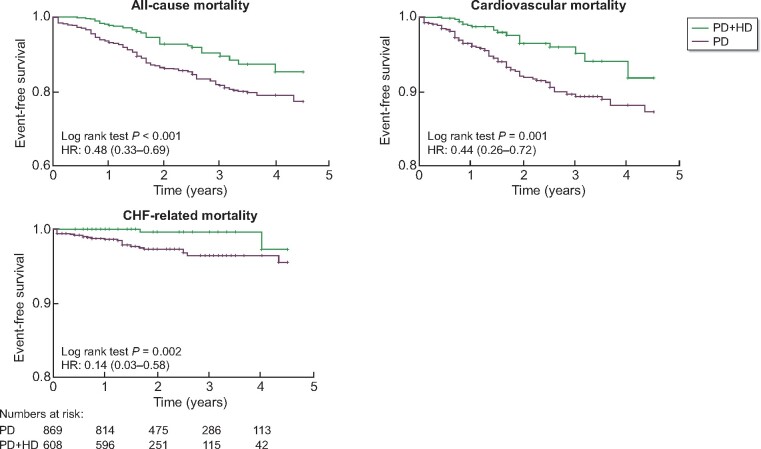
Comparison of all-cause mortality, CV mortality, CHF-related mortality before transition to HD among PS-matched cohort. The data were censored at the time of transition to HD. Time 0 indicated the time when combination therapy was started for PD + HD patients and the time of matching for PD alone patients. The numbers at risk are shown below the graphs. The numbers at risk were the same for all-cause, CV and CHF-related mortality. PD + HD, combination of PD with once-weekly HD.

The time to complete transition to HD (thrice-weekly HD without PD) was compared in the matched cohort. Complete transition to HD was significantly earlier for those on PD + HD therapy [HR 1.72 (1.45–2.03)] (Figure 2). The difference in time from matching to transition to HD between the groups was 0.37 (0.26–0.48) years. Lower mortality in the PD + HD group was possibly caused by better solute and fluid removal by earlier transition to HD. However, the HRs for mortality did not change when only events up to the time of complete transition to HD were considered (i.e. data were censored at the time of transition to HD) (Figure 4).

#### Sensitivity analyses

We performed additional matching on serum albumin and body weight (Supplementary data, Table S1) to eliminate the possibility that better nutritional status at baseline among PD + HD group leads to better outcomes (i.e. those with better nutritional status require higher ultrafiltration and might have selectively switched to PD + HD therapy). In this matching analysis, PD + HD therapy was still associated with significantly lower mortality [HR 0.45 (0.31–0.67), 0.34 (0.20–0.61) and 0.06 (0.01–0.41) for all-cause, CV and CHF-related mortality, respectively]. Imbalances in the causes of ESRD, urine output and history of myocardial infarction (MI) were seen among matched cohort, but adjustment for these variables did not substantially changed the results [HR 0.47 (0.32–0.69), 0.36 (0.21–0.64) and 0.05 (0.01–0.40) for all-cause, CV and CHF-related mortality, respectively].

Patients included in the PS-matched cohort belonged to 358 different dialysis facilities. Stratified Cox regression by PS-matched pairs or dialysis facilities yielded similar results (Table 2). Use of shared frailty models as dialysis facilities treated as a random effect did not change the results (Table 2). Analyses after exclusion of those who died on PD alone within 1 year (*n* = 74) and their matched pairs (*n* = 81) showed similar results (Table 2).

**Table 2.** Sensitivity analyses

	All-cause mortality	CV mortality	CHF-related mortality	Complete transition to HD
Cox regression	0.56	0.48	0.19	1.72
	(0.42–0.75)	(0.32–0.72)	(0.07–0.55)	(1.45–2.03)
Stratified Cox regression^a^				
By PS-matched pairs	0.57	0.52	0.20	1.90
	(0.42–0.77)	(0.34–0.79)	(0.07–0.56)	(1.58–2.29)
By dialysis facilities	0.54	0.48	0.34	1.71
	(0.37–0.80)	(0.28–0.80)	(0.11–1.06)	(1.36–2.17)
Shared frailty models^b^	0.55	0.48	0.19	1.73
	(0.40–0.74)	(0.32–0.73)	(0.07–0.56)	(1.45–2.07)
Exclusion of death <1 year on PD (*n* = 1322)^c^	0.63	0.50	0.29	
	(0.44–0.92)	(0.29–0.86)	(0.09–0.99)	

Data were shown as HRs (95% confidence intervals).

a Data were stratified by PS-matched pairs or dialysis facilities.

b Data were adjusted for quintiles of PS and dialysis facilities were treated as a random effect.

c Those who died on PD within 1 year and their matched pairs were excluded (*n* = 1322). The data were analysed by Cox regression analyses.

### Comparison of changes in body weight and daily urine volume

**FIGURE 5:**
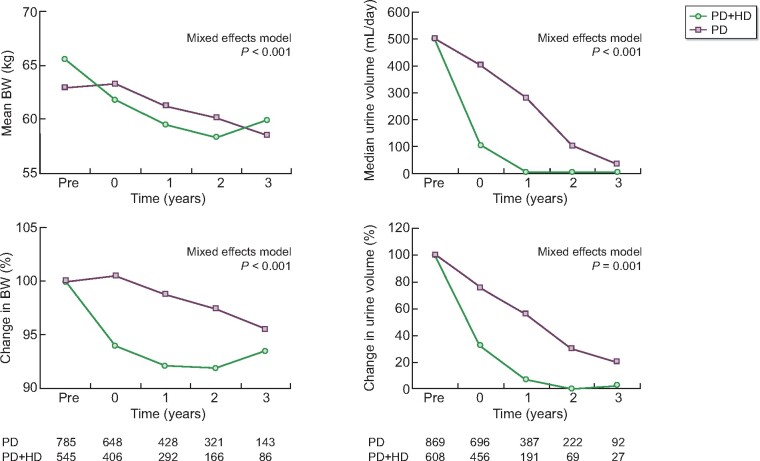
Comparison of body weight and urine volume between those who initiated PD + HD therapy and those who remained on PD alone. Numbers of patients with available data were shown below the graphs. Time 0 indicates the end of the year during which PD + HD therapy was initiated. BW, body weight; PD + HD, combination of PD with once-weekly HD.

Changes in body weight and daily urine volume were compared among the matched cohort (Figure 5). Reduction in body weight was more prominent among those on PD + HD therapy (P < 0.001 by mixed effects model). In addition, the decline in daily urine volume was more rapid among those on PD + HD therapy (P < 0.001 by mixed effects model). The results suggest that combination of once-weekly HD with PD improved fluid removal and that this may contribute to lower incidence of death due to CHF or CV events. On the contrary, decreased renal perfusion during HD might have hastened the loss of residual renal function.

## DISCUSSION

This longitudinal study showed that the decline in residual renal function was faster and complete transition to HD was earlier among those on combination of once-weekly HD with PD therapy compared with those on PD alone. On the contrary, combination therapy was associated with lower all-cause mortality, CV mortality and CHF-related mortality. HR for all-cause mortality was 0.56 (0.42–0.75) and comparable with that in a previous study at HR 0.60 (0.41–0.86) [4]. Number needed to treat to prevent one death per year was only 34 patients. This is of significant clinical importance. Decline in body weight and daily urine volume was more prominent in those on PD + HD therapy. These results suggest that lower all-cause mortality, CV mortality and CHF-related mortality might be due to better fluid removal by HD. In addition, better fluid removal by HD in PD + HD therapy might explain why the effect size for CHF-related mortality was the largest. At the same time, decreased renal perfusion during HD might have caused volume contraction and might have hastened the decline of residual renal function.

In this study, we used PS matching to eliminate indication bias as much as possible. As the most common reason to initiate PD + HD therapy was insufficient fluid and solute removal, patients were matched with factors affecting these decisions such as blood urea nitrogen, creatinine, PD vintage and daily urine volume. Patients were also matched with factors affecting outcomes such as age and history of CV events. The two groups were well balanced, and discrimination by the models from which PS was derived was reasonable. PD + HD therapy is almost exclusively performed in Japan, and no data have been available based on a large number of patients that could provide guidance on the optimal time to initiate this therapy or which patients most benefit from the therapy. As a result, some nephrologists start PD + HD therapy early after initiation of PD, believing that HD with little ultrafiltration in addition to PD preserves residual renal function and provides better nutritional status [13]. Many nephrologists utilize PD + HD therapy a few years following initiation [6, 8] of PD when they judge that fluid and solute removal was insufficient with PD alone, though other nephrologists keep patients on PD alone until complete transition to HD. The decisions to start PD + HD therapy depend on the preferences of patients and their treating physicians. These situations made it possible to perform PS matching between PD + HD therapy and PD alone.

In Figure 2, survival curves for PD + HD therapy and PD alone groups separated early (within 1 year). This might reflect the indication bias even after PS matching. For example, patients on PD and with limited life expectancy such as those with terminal malignancy are unlikely to initiate PD + HD therapy and there might be a bias towards worse survival in the PD alone group. Therefore, we excluded those on PD who died within 1 year and their matched pairs. This sensitivity analysis did not change the results substantially (Table 2).

Outcomes of PD patients could be influenced by dialysis facilities [14, 15]. Sensitivity analyses were performed using stratified Cox regression in which data were stratified by dialysis facilities and shared frailty models in which dialysis facilities were treated as a random effect. These sensitivity analyses yielded similar results. These results suggest that better outcomes among those on PD + HD therapy were not due to centre effects.

This study had several advantages over the previous study showing that PD + HD therapy was associated with lower all-cause mortality [4]. In the previous study, PD + HD therapy was treated as one of the independent variables in the Cox regression model. Although the data were adjusted for total dialysis vintage, it was unclear how long patients were on PD + HD therapy at baseline. In this study, those who initiated PD + HD therapy were matched with those on PD alone by the data just before the initiation of PD + HD therapy. Baseline characteristics were matched including PD vintage. Moreover, we performed vigorous sensitivity analyses considering centre effects or excluding early death among the PD group.

We had expected that the benefit of PD + HD therapy would be more prominent among younger patients or diabetic patients who were more at risk for volume overload and among those on PD for a few years with declining daily urine volume. However, the association between PD + HD therapy and all-cause mortality, CV mortality and CHF death were similar across age, PD vintage, diabetic status and baseline daily urine volume. These results suggest that even those who initiated PD with residual renal function might benefit from early use of PD + HD therapy. However, note that we matched patients who actually switched from PD to PD + HD therapy. It is still unclear whether we should start PD + HD therapy from the beginning of dialysis therapy.

To our knowledge, the strength of this study is that this is the largest cohort of PD + HD therapy. As PD + HD therapy is almost exclusively performed in Japan and JRDR covers more than 95% of the dialysis population in Japan, our study presented the best data about outcomes of PD + HD therapy. We also performed multiple rigorous sensitivity analyses to eliminate indication biases or centre effects as much as possible. The analyses excluding frail patients who died within 1 year on PD or analyses considering centre effects did not substantially change the results.

This study has limitations. As this is an observational study, there are possibilities of residual confounding. As JRDR is a survey performed at the end of each year, data at the end of the preceding year were used for PS matching, and it was unclear exactly when patients initiated PD + HD therapy within the given year. We had no data immediately before patients transitioned from PD alone to PD + HD therapy. There might have been changes in patient’s health status from the time of matching to the time when they actually initiated PD + HD therapy. Better survival in the PD + HD group might be due to better nutritional status and dialysis adequacy at baseline or after initiation of combination therapy. However, due to the large number of missing data, we could not perform matching on *Kt*/*V* or protein catabolic ratio (PCR). Moreover, for those on PD + HD therapy, no consensus exists on how to evaluate *Kt*/*V* or PCR, and it was unclear when these data were obtained in relation to the timing of HD therapy.

In conclusion, this study showed that PD + HD therapy was associated with lower all-cause mortality, CV mortality and CHF-related mortality, but earlier transition to HD compared with PD alone, which might be due to better fluid removal by HD. The use of PD + HD therapy might improve mortality while maintaining a flexible lifestyle with PD.

## SUPPLEMENTARY DATA

Supplementary data are available at ckj online.

## Supplementary Material

sfaa173_Supplementary_Data
