# Supplementary material for: Combination of once-weekly haemodialysis with peritoneal dialysis is associated with lower mortality compared with peritoneal dialysis alone: a longitudinal study
Source: Clin Kidney J. 2020 Nov 7;14(6):1610–7. doi: 10.1093/ckj/sfaa173 (PMC8162862; doi:10.1093/ckj/sfaa173)
Supplement: sfaa173_Supplementary_Data [file sfaa173_supplementary_data.docx]

Supplementary Table 1. Demographics of the propensity matched cohort

|  | PD+HD  (n=417) | PD  (n=588) | *d* (%) |
| --- | --- | --- | --- |
| age | 59.0 (11.8) | 59.0 (12.7) | 0.1 |
| Sex (male) | 292 (70.0) | 427 (72.6) | 5.7 |
| Causes of ESRD  Glomerulonephritis  Diabetes mellitus  Hypertension  Others | 173 (41.5)  127 (30.5)  49 (11.8)  68 (16.3) | 214 (36.4)  199 (33.8)  80 (13.6)  95 (16.1) | 10.5  -7.3  -5.6  0.4 |
| PD vintage (years)* | 2.1 (1.0-4.1) | 2.0 (1.0-4.0) | 8.3 |
| Blood urea nitrogen (mg/dL) | 58.1 (15.0) | 58.2 (15.6) | -0.3 |
| Creatinine (mg/dL) | 11.5 (2.9) | 11.3 (3.1) | 6.9 |
| Urine volume (mL/day) * | 490 (100-900) | 500 (250-1000) | -13.0 |
| History of myocardial infarction  Yes  No  Unknown | 23 (5.5)  359 (86.1)  35 (8.4) | 51 (8.6)  500 (85.0)  37 (6.3) | -12.3  2.4  8.1 |
| History of haemorrhagic stroke  Yes  No  Unknown | 6 (1.4)  377 (9.0)  34 (8.2) | 5 (0.9)  545 (9.3)  38 (6.5) | 5.5  -8.2  6.5 |
| History of ischemic stroke  Yes  No  Unknown | 43 (10.3)  341 (81.8)  33 (7.9) | 67 (11.4)  483 (82.1)  38 (6.5) | -3.5  -1.0  5.6 |
| History of limb amputation  Yes  No  Unknown | 3 (0.7)  382 (91.6)  32 (7.7) | 6 (1.0)  548 (93.2)  34 (5.8) | -3.2  -6.0  7.6 |
| Body weight (kg) | 64.2 (10.5) | 63.5 (12.2) | 5.8 |
| Albumin (g/dL) | 3.4 (0.5) | 3.4 (0.5) | -8.6 |

The data were shown in number (%), mean (SD), or median (interquartile range). PD+HD: combination of peritoneal dialysis with once weekly haemodialysis, PD: peritoneal dialysis, *d*: standardized difference, ESRD: end-stage renal disease

*Standardized differences were calculated after log-transformation.
